# Pantomime-Grasping: Advance Knowledge of Haptic Feedback Availability Supports an Absolute Visuo-Haptic Calibration

**DOI:** 10.3389/fnhum.2016.00197

**Published:** 2016-05-06

**Authors:** Shirin Davarpanah Jazi, Matthew Heath

**Affiliations:** ^1^School of Kinesiology, University of Western OntarioLondon, ON, Canada; ^2^Graduate Program in Neuroscience, University of Western OntarioLondon, ON, Canada

**Keywords:** action, grasping, haptic feedback, pantomime, Weber’s law

## Abstract

An emerging issue in movement neurosciences is whether haptic feedback influences the nature of the information supporting a simulated grasping response (i.e., pantomime-grasping). In particular, recent work by our group contrasted pantomime-grasping responses performed with (i.e., PH+ trials) and without (i.e., PH− trials) terminal haptic feedback in separate blocks of trials. Results showed that PH− trials were mediated via *relative* visual information. In contrast, PH+ trials showed evidence of an *absolute* visuo-haptic calibration—a finding attributed to an error signal derived from a comparison between expected and actual haptic feedback (i.e., an internal forward model). The present study examined whether advanced knowledge of haptic feedback availability influences the aforementioned calibration process. To that end, PH− and PH+ trials were completed in separate blocks (i.e., the feedback schedule used in our group’s previous study) and a block wherein PH− and PH+ trials were randomly interleaved on a trial-by-trial basis (i.e., random feedback schedule). In other words, the random feedback schedule precluded participants from predicting whether haptic feedback would be available at the movement goal location. We computed just-noticeable-difference (JND) values to determine whether responses adhered to, or violated, the relative psychophysical principles of Weber’s law. Results for the blocked feedback schedule replicated our group’s previous work, whereas in the random feedback schedule PH− *and* PH+ trials were supported via relative visual information. Accordingly, we propose that *a priori* knowledge of haptic feedback is necessary to support an absolute visuo-haptic calibration. Moreover, our results demonstrate that the *presence* and *expectancy* of haptic feedback is an important consideration in contrasting the behavioral and neural properties of natural and simulated grasping.

## Introduction

Our visual system’s ability to identify an object is dependent on the integration of *relative* information laid down and maintained by the visuoperceptual networks of the ventral visual pathway. In contrast, goal-directed grasping is supported by *absolute* visual information mediated by dedicated visuomotor networks residing in the posterior parietal cortex (PPC) of the dorsal visual pathway (for reviews of duplex visual processing see Goodale, 2011; Whitwell et al., 2014)^1^. The importance of vision for action and the absolute processing of the dorsal visual pathway is characterized by work showing that chronic (i.e., optic ataxia; for recent review see Andersen et al., 2014) and transient (i.e., via transcranial magnetic stimulation) lesions to the PPC impairs grip aperture scaling and interferes with online trajectory amendments (Jeannerod, 1986; Desmurget et al., 1999; Pisella et al., 2000; Cavina-Pratesi et al., 2013). It is, however, important to recognize that in addition to vision, the motor system is provided object-based information via haptic feedback (i.e., integrative mechano- and proprioceptive cues). In particular, physically grasping an object provides: (1) mechanoreceptive cues related to the shape and texture of an object’s grasp points; and (2) proprioceptive cues from thumb and forefinger position that provide absolute object size information (for review of haptic frames of reference see Lederman and Klatzky, 2009). As such, haptic feedback may serve as an important sensory resource in determining the nature of the information (i.e., relative vs. absolute) supporting grasping control.

One area of research that has potentially underestimated the importance of haptic feedback is pantomime-grasping. The empirical evaluation of pantomimed (or simulated) actions was first introduced by Liepmann (1905/1980) and required that individuals perform a well-learned movement (e.g., hammering a nail) in the absence of a physical tool and/or object. The task was originally employed to provide clinical evaluation of apraxic motor deficits following stroke (Geschwind and Kaplan, 1962; Roy et al., 2000). The grasping literature has subsequently evolved the use of pantomime-grasping and requires that participants direct a response to an area adjacent to, or once occupied by, a target object. In particular, the dissociated stimulus-response relations of pantomime-grasping have been frequently used as a framework for understanding the distinct *visual* characteristics associated with natural and simulated responses (for review see Goodale, 2011). It is, however, important to recognize that pantomime-grasping and natural grasping differ not only in terms of their visual properties but also because the former does not entail physically interacting with an object; that is, pantomime-grasping does not afford the integration of haptic feedback. In addressing the importance of this issue, Bingham et al. (2007) employed a mirror-box apparatus allowing the manipulation of haptic feedback without occluding object vision (see depiction of mirror-box in Figure 1 of Bingham et al., 2007). In that experiment, responses were completed in conditions wherein vision of an object overlapped with its physical location (i.e., haptic feedback condition: H+ trials) and when the physical object was unavailable at the movement goal location (i.e., no haptic feedback condition). Thus, the no haptic feedback condition in Bingham et al.’s (2007) study entailed a pantomime action and we henceforth refer to this condition as *pantomime-grasping without haptic feedback* (i.e., PH− trials). Notably, H+ and PH− trials were completed in separate blocks (i.e., blocked feedback schedule) and a block wherein task-types were randomly interleaved on a trial-by-trial basis (i.e., random feedback schedule). Thus, in the random feedback schedule participants were unaware as to whether they would receive haptic feedback at the end of their response. Blocked feedback schedule PH− trials exhibited a less accurate scaling of grip aperture to object size (i.e., smaller peak and terminal grip aperture values) than H+ trials. In contrast, random feedback schedule PH− trials exhibited aperture scaling commensurate to random and blocked schedule H+ trials. Accordingly, Bingham et al. (2007) concluded that haptic feedback—even when intermittently and unpredictably available—supports an absolute visuo-haptic calibration. In contrast, the absence of haptic feedback throughout a block of trials (i.e., blocked PH− trials) was interpreted to preclude any calibration and limit grip aperture specification to the relative visual (i.e., visuoperceptual) properties of an object (see also Goodale et al., 1994; Westwood et al., 2000; Cavina-Pratesi et al., 2011; Fukui and Inui, 2013; Holmes et al., 2013). In subsequent work, Schenk (2012) used a similar mirror-box apparatus to examine H+ and PH− trial performance in an individual with bilateral lesions to her ventral visual pathway (i.e., patient DF; see James et al., 2003). The literature has shown that DF’s ventral stream lesions impair her visual form perception but spare her use of vision for action due to her intact dorsal visual pathway (Goodale and Milner, 2006). Schenk reported that DF’s grip aperture specification during PH− trials was no better than her well-documented visuoperceptual deficits—a finding previously documented and attributed to the relative and perception-based nature of pantomime-grasping (Goodale et al., 1994). In turn, DF demonstrated absolute aperture scaling when PH− trials were performed in a feedback schedule that included intermittent—but predictably available—H+ trials^2^. Based on these results, Schenk proposed that DF requires integrative visual and haptic cues to support her absolute aperture scaling. Although Schenk did not provide a mechanistic account for his findings, Whitwell et al. (2014) proposed that *if* haptic feedback supports DF’s grip aperture scaling then it may do so by providing feedback related to thumb and index finger position that is used in a feedforward fashion to support performance on future trials, and/or generate an error signal that permits an absolute visuo-haptic calibration (for challenges to Schenk’s findings and interpretation see Whitwell and Buckingham, 2013; Whitwell et al., 2014; see also Milner et al., 2012).

Recent work by our group showed that dissociable information supports grasping responses performed with and without haptic feedback (Davarpanah Jazi et al., 2015a,b; Hosang et al., 2016; see also Davarpanah Jazi and Heath, 2014). Notably, a distinction between our group’s work and others (Bingham et al., 2007; Schenk, 2012) is that instead of contrasting PH− and H+ trials our group employed a pantomime-grasping condition wherein haptic feedback was provided after participants achieved their desired movement goal location (henceforth referred to as *pantomime-grasping with haptic feedback*: PH+). In particular, PH+ trials entailed an experimenter placing a physical object between participants’ thumb and forefinger only after their grasping response was completed. Thus, and unlike H+ trials, PH+ trials provided: (1) no expectancy that the object would be available to grasp immediately at the end of the response; and (2) no risk of an early object collision (see Smeets and Brenner, 1999). For example, the PH− and PH+ trials employed by Davarpanah Jazi et al. (2015b) were completed in separate blocks. Additionally, just-noticeable-difference (JND) values at the time of peak grip aperture (PGA) were calculated to determine whether task-types adhered to, or violated, the psychophysical principles of Weber’s law. Indeed, Weber’s law asserts that the JNDs associated with discriminating between an original (i.e., the to-be-grasped target object) and a comparator stimulus (i.e., grip aperture) is in constant proportion to the magnitude of the original stimulus, and that the sensitivity of detecting a change in any physical continuum is *relative* as opposed to *absolute* (for review of this issue in grasping, see Heath et al., 2015a). As such, JNDs in grasping provide a law-based evaluation of the nature of the information supporting motor output (see Ganel et al., 2008a; Heath et al., 2011). Results showed that JNDs for the PH− and PH+ trials adhered to and violated Weber’s law, respectively. In line with previous work, results for the PH− trials indicated aperture shaping via relative visual information (e.g., Goodale et al., 1994; Bingham et al., 2007; Cavina-Pratesi et al., 2011; Holmes et al., 2013). In turn, that PH+ trials violated Weber’s law indicates that the provision of haptic feedback supports the absolute specification of object size. More specifically, our group proposed that PH+ trials engender an error signal related to a difference between an “expected” (in this case haptic) and “actual” sensory outcome that supports an absolute visuo-haptic calibration mediating future trials (for review of internal models see Wolpert et al., 1995). Indeed, such a view is consistent with evidence that haptic feedback is as a salient “intermodal alignment” signal that supports the learning and the predictions necessary for future motor responses (Flanagan et al., 2006).

The goal of the present investigation was to examine the issue of whether advanced knowledge related to the provision of haptic feedback influences the information supporting PH− and PH+ trials. The basis for our question was twofold. First, and as mentioned above, it is possible that the PH− trials used in Bingham et al.’s (2007) random feedback schedule were influenced by an expectation that the object would be available at the movement goal location. Indeed, because Bingham et al.’s random feedback schedule included PH− and H+ trials it is entirely possible that participants structured their responses based on a strategy designed to avoid colliding with the object in the event that it was present. In fact, the authors of that work acknowledge that such a strategy may account for the equivalent peak and terminal grip aperture values associated with their random feedback schedule PH− and H+ trials. To that end, we contrasted PH− and PH+ trials performed in blocked (i.e., the same feedback schedule as used by Davarpanah Jazi et al., 2015b) and random feedback schedules. Importantly, the use of PH− and PH+ trials in the random feedback schedule provides equivalent movement strategies because the absence of a physical object in both tasks obviates the need for responses to be structured as if the object was always available to touch, or collide with. Second, we computed JNDs across all experimental conditions to provide a law-based measure of whether advance knowledge related to the provision of haptic feedback influences the information supporting grasping. In terms of research predictions, if PH+ trials in the random feedback schedule violate Weber’s law then results would support the contention that intermittent—and unpredictable—haptic feedback is sufficient to support an absolute visuo-haptic calibration. In turn, if PH+ trials in the random feedback schedule adhere to Weber’s law then results would indicate that advanced knowledge of haptic feedback availability is necessary to support an absolute visuo-haptic calibration. Moreover, evidence supporting the latter view would indicate that the inability to contrast actual and expected haptic events on a trial-by-trial basis precludes the development of an internal model necessary for the aforementioned calibration. In addition, we included memory-guided (MG) trials wherein haptic feedback was immediately available at the movement goal location. The MG trials were employed as a naturalistic control for the integration of haptic feedback.

## Materials and Methods

### Participants

Sixteen individuals (1 male and 15 females: age range = 18–29 years) from the University of Western Ontario community volunteered to participate in this study. All participants were self-declared right hand dominant and had normal or corrected-to-normal vision. Participants signed consent forms approved by the Office of Research Ethics, University of Western Ontario, and this work was completed according to the Declaration of Helsinki. The participants recruited here were a convenience sample and we recognize that it resulted in an asymmetrical number of female participants. That said, a previous study by our group reported null sex-based differences in the integration of haptic feedback for grasping kinematics (Davarpanah Jazi and Heath, 2014). Thus, we do not believe that our results are tempered by sex-based differences in grasping control.

### Apparatus and Procedures

**Figure 1:**
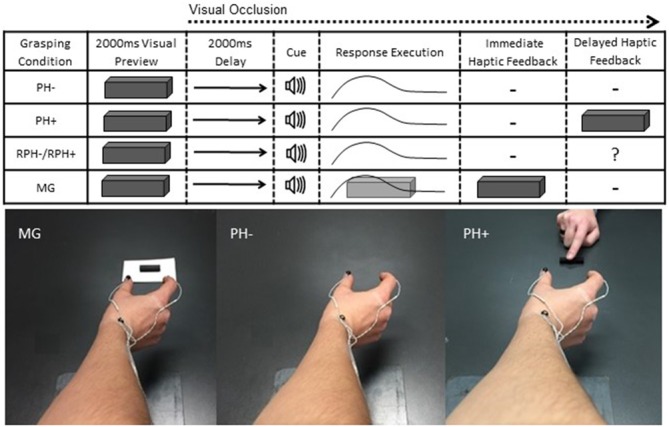
**Schematic of visual, auditory and haptic events for the memory-guided (MG) and pantomime-grasping trials with (i.e., PH+) and without (i.e., PH−) haptic feedback in blocked (i.e., BPH− and BPH+) and random (RPH− and RPH+) feedback schedules.** Participants were provided a 2000 ms visual preview of a target object 20, 30, 40, or 50 (mm) in width after which time vision was occluded for a 2000 ms delay and followed by an auditory tone. For MG trials, the target object remained present on the grasping surface and therefore provided immediate haptic feedback. For the pantomime-grasping trials, the target object was removed from the grasping environment and was not available to “touch” at the movement goal location. At the end of BPH+ and RPH+ trials the experimenter placed the physical target object between participants’ thumb and forefinger to provide delayed haptic feedback. For RPH− and RPH+ trials, the *question mark* in the column headed “*Delayed Haptic Feedback*” indicates that participants were unaware of whether haptic feedback would be available. The photographs below the schematic provide an egocentric view of a participant’s limb position at the movement goal location for MG, PH− and PH+ trials. Notably, for the PH+ trials the experimenter’s limb can also be seen placing the target object between the participant’s thumb and forefinger. Note: the goggles were in their translucent state throughout a movement; hence, the egocentric view presented here serves only to depict participants’ grasp posture.

Participants stood in front of a table-top (height = 880 mm, depth = 760 mm, width = 1060 mm) for the duration of the experiment and used a precision grip (i.e., thumb and forefinger) to grasp the long axis of differently sized target objects with their right hand (see Figure 1 for grasping posture). The target objects were black acrylic blocks that were different in width (20, 30, 40 and 50 mm) but had the same height and depth (10 mm). Target objects were positioned 300 mm from the front edge of the table and at participants’ midline. The target objects’ long axis was oriented perpendicular to participants’ midline. A pressure sensitive switch placed at table midline and 50 mm from the front edge of the table served as the start location for each trial. Vision of the grasping environment was controlled via liquid-crystal shutter goggles (PLATO Translucent Technologies, Toronto, ON, Canada; for further information see: Milgram, 1987). As well, a Sonalart (Mallory Sonalert Products, Indianapolis, IN, USA) was used to cue grasping responses. Computer and auditory events were controlled via MATLAB (7.9.0: The MathWorks, Natick, MA, USA) and the Psychophysics toolbox extensions (ver 3.0; Brainard, 1997). A National Instruments A/D board (NI PCI-6221, National Instruments, Austin, TX, USA) supported external hardware connections (i.e., start location switch, translucent goggles, and Sonalert).

### Memory-Guided Grasping

Prior to each trial the experimenter placed a target object on the tabletop surface while the participant rested the medial surface of their grasping limb on the start location—during this time the goggles were set to their translucent state. Once the target was appropriately placed, the goggles were set to their transparent state for a 2000 ms visual preview. Following the preview the goggles reverted to their translucent state for a 2000 ms delay interval after which time a tone (2900 Hz for 100 ms) cued participants to initiate a grasping response. Participants were instructed to grasp—but not lift—and hold the target object for 2000 ms before returning to the start location. The goggles remained translucent for the duration of the response, thus participants planned and executed their response in the absence of online visual feedback. Notably, the target object remained on the table surface for the duration of the response and provided immediate terminal haptic feedback related to absolute object size. The MG condition was performed in a single block of trials and participants were therefore aware that a physical target object would be present at the movement goal location.

### Pantomime Grasping

Participants completed two types of pantomime-grasping trials and both entailed the same visual and auditory events as the MG task. In particular, the *pantomime-grasping without haptic feedback* trials (PH−) served as a more “traditional” pantomime-grasping response and involved the experimenter removing the target object from the grasping environment during the delay interval. As such, participants grasped to a remembered target location and were not afforded terminal haptic feedback related to object size. Further, participants were instructed to maintain their terminal aperture for 2000 ms before returning to the start location. In the *pantomime-grasping with haptic feedback* trials (PH+), the experimenter removed the target object from the grasping environment as in the PH− trials; however, after movement offset (see kinematic definition of movement offset below) the experimenter placed the target object between participants’ right thumb and forefinger. More specifically, a tone generated via the kinematic defined movement offset signaled the experimenter to place the target object back on the table surface and the experimenter slid the object until it first contacted the thumb and then positioned the object until the opposite side contacted the forefinger of the grasping hand. The time required to complete this process was not longer than 2500 ms. Notably, this time window has been shown to be sufficiently brief to allow for feedback-based integration (for review see Heath et al., 2010). Participants were then instructed to make the appropriate adjustments to produce a stable precision grasp (i.e., a forefinger and thumb posture that would allow for lifting of the target object). The target object was held—but not lifted—for 2000 ms before the participant returned to the start location. Figure 1 provides a schematic representation of the sequence of visual, auditory and haptic events that occurred during a single trial across all task-types. The 2000 ms visual delay between target preview and response cuing provided the experimenter with sufficient time to remove the target object from the table-top during pantomime-grasping trials. Further, previous work by our group has shown that MG grasping movements (i.e., the control condition in this experiment) completed following a delay (of 2000 ms or less) violate Weber’s law and are mediated via absolute visual information—a finding we have replicated on a number of occasions (Holmes et al., 2011; Davarpanah Jazi et al., 2015b; Hosang et al., 2016; for review see Heath et al., 2015a). Thus, our group has shown that the delay interval used here does not influence the nature of the information mediating motor output. Further, and in line with our group’s previous work (Davarpanah Jazi et al., 2015b), MG, PH− and PH+ trials were completed in a 600–800 ms grasping time bandwidth. Following each trial verbal feedback (i.e., “too fast”, “too slow”, “good”) was provided, and any trial falling outside the bandwidth was discarded and reentered into the trial matrix. Less than 5% of trials were repeated for this reason.

PH− and PH+ trials were performed in *blocked* (i.e., BPH− and BPH+) and *random* (i.e., RPH− and RPH+) feedback schedules. In the blocked feedback schedule (i.e., the same feedback schedule as used by Davarpanah Jazi et al., 2015b) participants were aware of whether or not terminal haptic feedback would be available, whereas in the random feedback schedule the presence of such feedback could not be predicted. More specifically, in the random feedback schedule PH+ and PH− trials were randomly interleaved on a trial-by-trial basis. The different trial blocks entailed 15 trials to each object size (which were randomly ordered). Therefore, the MG, BPH− and BPH+ trial blocks each consisted of 60 trials and each required approximately 30 min to complete. In turn, the random feedback schedule entailed 120 trials (i.e., 60 trials of each of the RPH− and RPH+ tasks) and required approximately 60 min to complete. To reduce mental and physical fatigue, the four trial blocks were performed in separate sessions separated by at least 24 h (i.e., two blocks per session). The ordering of trial blocks was randomized.

### Data Analysis

The position of the right limb was measured via infrared emitting diodes (IREDs) placed on the lateral surface of the distal phalanx of the forefinger, the medial surface of the distal phalanx of the thumb, and the styloid process of the wrist. IRED position data were sampled at 400 Hz via an OPTOTRAK Certus for 1500 ms following response cuing. IRED position data were filtered offline via a second-order dual-pass Butterworth filter employing a low-pass cutoff frequency of 15 Hz (for further information see Winter and Patla, 1997). Subsequently, instantaneous velocities were computed from the position (i.e., displacement) data via five-point central finite difference algorithm. Movement onset was marked when participants released pressure from the start location switch and movement offset was determined when wrist velocity fell below a value of 50 mm/s for 20 consecutive frames (i.e., 50 ms).

### Just-Noticeable-Difference (JND) Values

**Figure 2:**
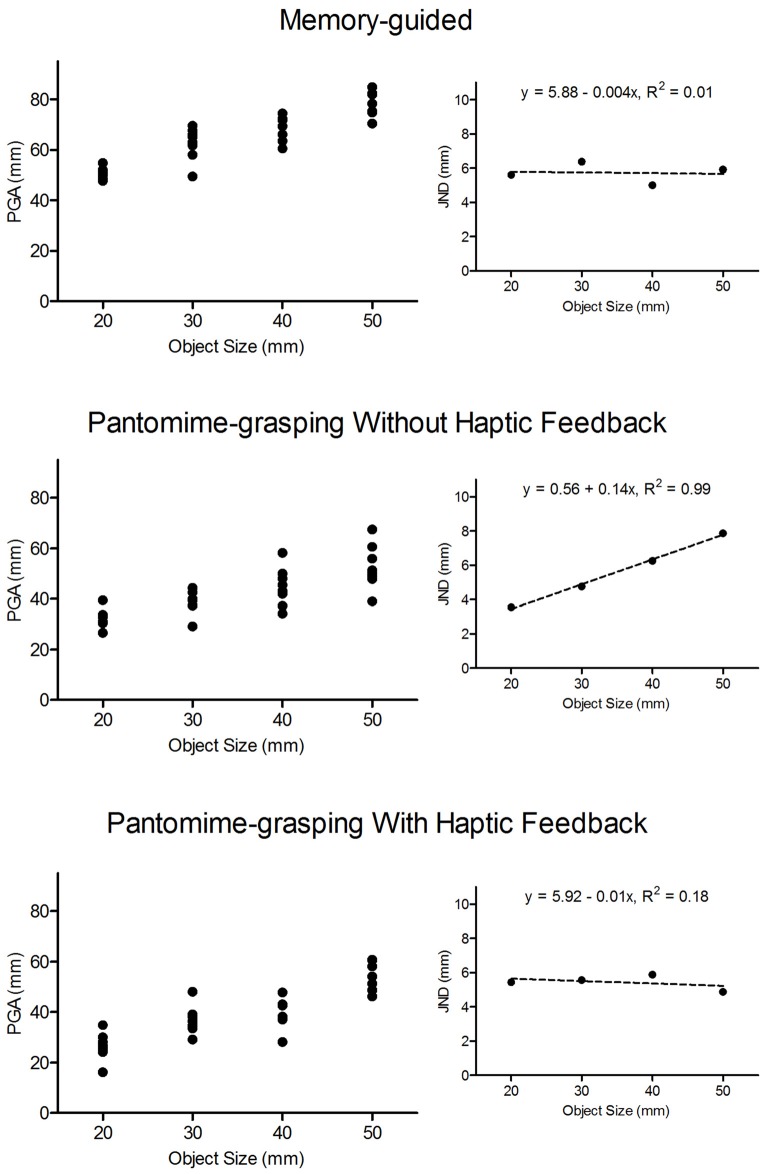
**Trial-to-trial peak grip aperture (PGA: in mm) values for an exemplar participant in MG (top panel), blocked feedback schedule pantomime-grasping without haptic feedback (BPH−: middle panel) and blocked feedback schedule pantomime-grasping with haptic feedback (BPH+: bottom panel) trials as a function of object size.** The figure demonstrates that trial-to-trial PGAs for MG and BPH+ trials did not systematically vary with object size, whereas values for BPH− trials increased with increasing object size. The smaller offset panels represent the mean within-participant standard deviation for each object size (i.e., the just-noticeable-difference values: JNDs). The dashed lines represents the linear regression of JNDs to object size and the top of each panel presents the associated linear regression equation and proportion of explained variance. The figure graphically demonstrates our computation and interpretation of JNDs. In particular, null scaling of JNDs to object size (i.e., MG and BPH+ trials) is taken as a violation of Weber’s law, whereas values that systematically increase with object size (i.e., BPH− trials) are taken as adherence to the law.

Weber’s law asserts that JNDs represent the smallest detectable difference between an original and a comparator stimulus and are proportional to the magnitude of the original stimulus. Moreover, the law states that the sensitivity of detecting a change in any physical continuum is relative as opposed to absolute. In the perceptual literature JNDs are computed via an arbitrary statistical criterion related to participants’ ability to discriminate between an original and a comparator stimulus (e.g., 75% of trials or any other possible value). Notably, however, a statistical criterion is not possible for a grasping task. Thus, in the current and other research (Ganel et al., 2008a,b; Pettypiece et al., 2009; Holmes et al., 2011, 2013; Heath et al., 2012; Holmes and Heath, 2013; Davarpanah Jazi and Heath, 2014; Davarpanah Jazi et al., 2015b) JNDs represent the within-participants standard deviation of PGA. In addition, we computed JNDs at movement offset (i.e., terminal grip aperture: TGA) to evaluate whether a visuo-haptic calibration extends from the predictive (i.e., PGA; see Jeannerod, 1986) to the end stage of aperture shaping. Importantly, the JND approach used here is based on the Fechnerian principle that variance reflects the uncertainty by which a performer is unable to detect a difference between an original and comparator stimulus (Ganel et al., 2008a; Heath et al., 2015a; for extensive review see Marks and Algom, 1998). In particular, Marks and Algom assert that a linear increase in variability with increasing stimulus intensity “… is Weber’s law” (p. 102). Figure 2 provides data from an exemplar participant performing MG, BPH− and BPH+ trials. The large panels show trial-to-trial PGAs associated with 20, 30, 40 and 50 mm target objects. Further, the offset panels of Figure 2 show standard deviations (i.e., JNDs) associated with the trial-to-trial values. The figure shows that trial-to-trial values for the BPH− trials—but not MG or BPH+ trials—increased linearly with increasing object size and we interpret the linear increase as adherence to Weber’s law.

### Dependent Variables and Statistical Analyses

In line with our previous work, we examined grasping time (GT: time between movement onset and offset), peak grip aperture (PGA: maximum resultant distance between thumb and forefinger), terminal grip aperture (TGA: distance between thumb and forefinger at movement offset), time to peak grip aperture (tPGA: time from movement onset to PGA) and computed JNDs at PGA and TGA. All dependent variables were examined via 5 (condition: MG, BPH−, BPH+, RPH− and RPH+) by 4 (object size: 20, 30, 40, and 50 mm) repeated measures ANOVA. Main effects and interactions were considered significant at an alpha level of 0.05 or less. *Post hoc* contrasts for object size were examined via power-polynomials (i.e., trend analysis: see Pedhazur, 1997), whereas between-condition effects were decomposed via paired samples *t*-tests. We also computed participant-specific slopes relating JNDs (at PGA and TGA) to object size across the five grasping conditions (i.e., MG, BPH−, BPH+, RPH− and RPH+). The slope analyses were designed to support a series of planned contrasts. The first examined all pairwise comparisons between MG, BPH− and BPH+ trials, whereas the second examined all pairwise comparisons between BPH−, RPH− and RPH+ trials. The basis for these analyses was to: (1) determine whether advance knowledge of haptic feedback in a pantomime-grasping task (i.e., BPH+ trials) elicits a null JND/object size scaling commensurate to a more naturalistic grasping task (i.e., MG trials); and (2) determine whether the absence of advance haptic feedback information (i.e., RPH− and RPH+ trials) renders aperture scaling commensurate to a “traditional” pantomime-grasping task (i.e., BPH− trials).

## Results

**Figure 3:**
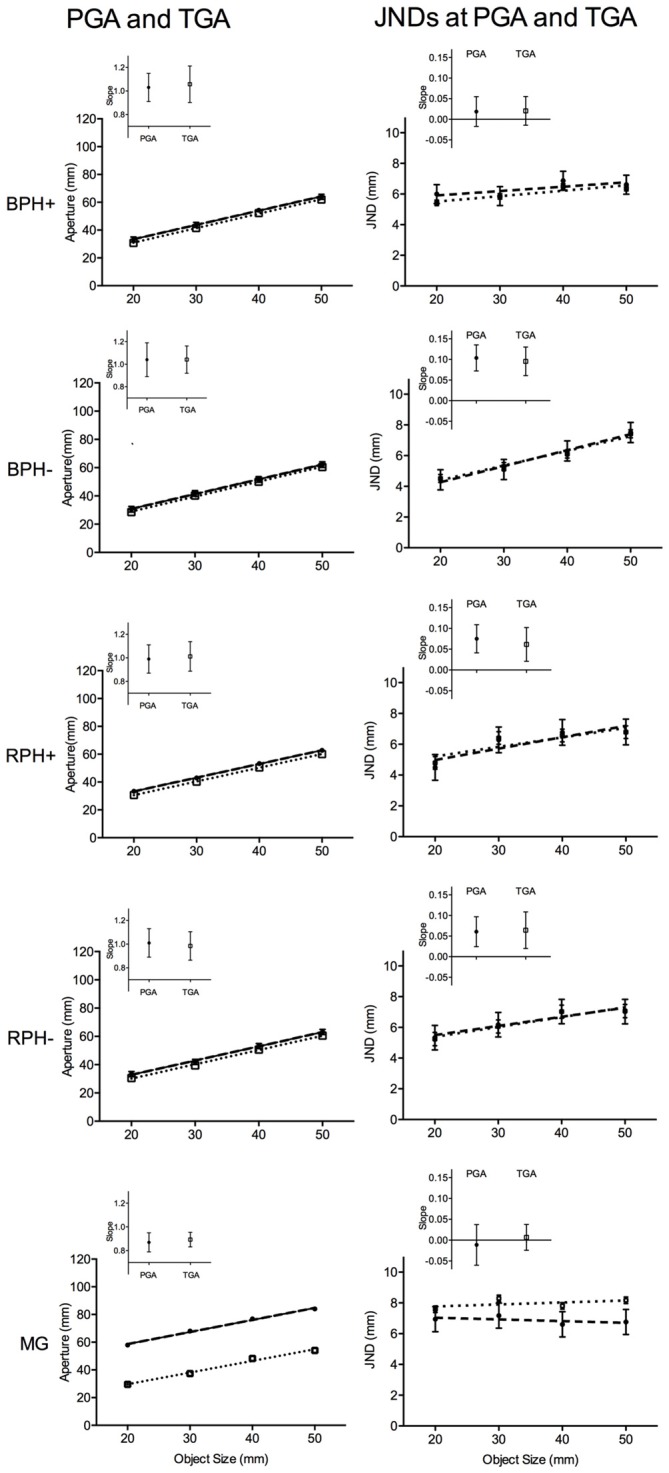
**The left panels present mean peak grip aperture (PGA in mm; see closed circle symbols and dashed regression line) and terminal grip aperture (TGA in mm; see open square symbols and dotted regression line) and the right panels depict just-noticeable-difference (JND in mm) values computed at PGA (see closed circle symbols and dashed regression line) and TGA (see open square symbols and dotted regression line) for: (1) blocked feedback schedule pantomime-grasping trials performed with (BPH+) and (2) without (BPH−) haptic feedback, and (3) random feedback schedule pantomime-grasping trials performed with (RPH+) and (4) without** **(RPH−) haptic feedback, and (5) MG trials.** Error bars represent 95% within-participants confidence intervals as a function of the mean-squared error term for object size computed separately for each trial-type (Loftus and Masson, 1994). Note: for the PGA and TGA panels the size of the error bars is less than the width of the symbol depicting the mean value, as well, for all pantomime-grasp trial-types a significant degree of overlap exists between PGA and TGA values. The inset panels represent the mean participant-specific slope relating PGA and TGA to object size and JNDs (computed separately at PGA and TGA) to object size. Error bars represent 95% between-participant confidence intervals and the absence of overlap between a confidence interval and the horizontal line indicates that the slope reliably differed from zero, and is a result that can be interpreted inclusive to a test of the null hypothesis (Cumming, 2013).

The average GT was 693 ms (SD = 27) and this variable did not produce any manipulation related effects (all *F* < 1). Results for tPGA yielded main effects for condition, *F*_(4,60)_ = 26.76, *p* < 0.001, and object size, *F*_(3,45)_ = 7.46, *p* < 0.01. In particular, tPGA values for pantomime-grasping conditions did not reliably vary (BPH− = 599 ms, SD = 46; BPH+ = 609 ms, SD = 36; RPH− = 574 ms, SD = 39; RPH+ = 578 ms, SD = 40; all *t*_(15)_ < 1) and occurred later than the MG condition (507 ms, SD = 45; all *t*_(15)_ > 5.80, all *p* < 0.001). In addition, across all trial-types tPGA increased linearly with increasing object size (only linear effect significant: *F*_(1,15)_ = 9.44, *p* < 0.01). Results for PGA produced main effects for condition, *F*_(4,60)_ = 31.82, *p* < 0.001, object size, *F*_(3,45)_ = 399.19, *p* < 0.001, and their interaction, *F*_(12,180)_ = 2.76, *p* < 0.01. Figure 3 shows that PGAs for all trial-types increased with increasing object size (only linear effects significant: all *F*_(1,15)_ = 206.79, 338.38, 207.78, 355.77 and 328.93 for BPH−, BPH+, RPH−, RPH+ and MG trials, respectively, all *p* < 0.001). As well, at each matched object size PGAs for the MG condition were larger than all pantomime trial-types (all *t*_(15)_ > 4.91, all *p* < 0.001), which did not reliably differ from one another (all *t* < 1). In terms of TGA, results indicated a main effect for object size, *F*_(3,45)_ = 428.94, *p* < 0.001, such that values increased linearly with increasing object size (only linear effect significant: *F*_(1,15)_ = 514.16, *p* < 0.001; see Figure 3). As well, we note that the absence of a reliable effect of condition, *F*_(4,60)_ < 1, for TGA demonstrates that the larger aperture values associated with MG trials early in the grasping trajectory (i.e., at PGA) were no longer present at movement offset.

Results for JNDs computed at PGA and TGA yielded main effects of object size, all *F*_(3,45)_ = 11.26 and 11.01 for JNDs at PGA and TGA, respectively, all *p* < 0.001, and condition by object size interactions, all *F*_(12,180)_ = 3.49 and 2.70 for JNDs at PGA and TGA, respectively, all *p* < 0.01. Figure 3 demonstrates that JNDs computed at PGA for BPH−, RPH− and RPH+ trials increased linearly with increasing object size (only linear effects significant: all *F*_(1,15)_ = 50.63, 12.02 and 21.63 for BPH−, RPH− and RPH+ conditions, respectively, all *p* < 0.001), whereas JNDs for the BPH+ and MG conditions did not reliably vary with object size, all *F*_(3, 45)_ = 1.46 and 0.36, *p*s = 0.24 and 0.78. In addition, JNDs computed at TGA match the aforementioned analyses; that is, values for BPH−, RPH− and RPH+ conditions increased linearly with increasing object size (only linear effects significant: all *F*_(1,15)_ = 14.75, 10.40, and 9.52 for BPH−, RPH− and RPH+ conditions, respectively, all *p* < 0.01), whereas BPH+ and MG conditions did not reliably vary with object size, all *F*_(3,45)_ = 1.72 and 0.08 for BPH+ and MG conditions, respectively, *p*s = 0.17 and 0.50.

The inset panels for JNDs in Figure 3 provide mean JND/object size slopes (for values computed at PGA and TGA) and associated 95% confidence intervals for each trial-type. These figures graphically support our analyses of mean JND values; that is, slopes for the BPH−, RPH+ and RPH− trials—but not the BPH+ and MG trials—reliably differed from zero. As well, we used participant-specific slopes for a series of planned comparisons and for ease of presentation we present here only JND/object size slopes computed at the time of PGA.^3^ The first set of planned comparisons show that the slope for BPH− trials (0.10, SD = 0.06) was steeper than MG (−0.01, SD = 0.09) and BPH+ trials (0.02, SD = 0.05; all *t*_(15)_ = 4.37 and 4.05, all *p* < 0.002), and the latter two trial-types did not reliably differ (*t*_(15)_ = −1.22, *p* = 0.24). A second set of planned comparisons indicated that RPH− (0.06, SD = 0.07), RPH+ (0.08, SD = 0.06) and BPH− trials did not reliably differ from one another (all *t*_(15)_ = 1.65, 1.31, and −1.00, all *p* > 0.33, respectively for RPH− vs. BPH−, RPH+ vs. BPH−, and RPH− vs. RPH+).

## Discussion

Our group demonstrated previously that PH− and PH+ trials performed in separate blocks adhere to, and violate Weber’s law, respectively. This demonstrates that haptic feedback supports an absolute visuo-haptic calibration (Davarpanah Jazi et al., 2015b). The present study contrasted PH− and PH+ trials across blocked and random feedback schedules to determine whether advanced knowledge of haptic feedback is necessary to support the aforementioned calibration.

### Memory-Guided (MG) and Blocked Feedback Schedule Pantomime-Grasping with (BPH+) and without (BPH−) Haptic Feedback

We first outline findings for MG and blocked pantomime-grasping trials (i.e., BPH− and BPH+) to demonstrate that results replicate an earlier study by our group (Davarpanah Jazi et al., 2015b). In particular, PGA and tPGA values for MG, BPH− and BPH+ trials increased linearly with increasing object size—a finding demonstrating that the motor system reliably discriminated between the differently sized objects used here (for resolution of visuomotor system see Ganel et al., 2012). Notably, however, MG trials produced larger and earlier occurring PGAs than BPH− and BPH+ trials (Cavina-Pratesi et al., 2011; Holmes et al., 2013; Davarpanah Jazi et al., 2015b; see also Goodale et al., 1994; Westwood et al., 2000). These results are consistent with previous work and demonstrate that the absence of a physical object (i.e., BPH− and BPH+ trials) offers no risk of an object “collision” and thereby renders PGA values that are smaller and later occurring than MG trials (for review of double-pointing hypothesis see Smeets and Brenner, 1999). In further support of this assertion, MG trials produced comparable terminal grip apertures (i.e., TGA) to BPH− and BPH+ trials—a result further indicating that the larger PGA of MG trials is related to an obligatory strategy designed to reduce the possibility of a collision. More notably, the timing and magnitude of PGAs, as well as the magnitude of TGA, for BPH− and BPH+ trials did not differ. This is a salient finding for two reasons. First, it demonstrates that trial-types were associated with comparable movement strategies. Second, it demonstrates that any difference in JND values across BPH− and BPH+ trials (see details below) cannot be attributed to a range effect in aperture size (i.e., larger JND for a response with a larger PGA or TGA; Lemay and Proteau, 2001) or the stochastic properties of motor-output variability (Schmidt et al., 1979)^4^.

We computed JNDs at the time of PGA and TGA to provide a law-based measure of whether MG, BPH−, and BPH+ trials adhere to or violate Weber’s law. Results for JNDs computed at PGA and TGA matched one another and showed that BPH− trials adhered to Weber’s law, whereas MG and BPH+ trials violated the law. Further, the mean JND/object size slope for BPH− trials was larger than MG and BPH+ trials, and the latter two trial-types did not differ. These findings provide a direct replication of our group’s previous work (Davarpanah Jazi et al., 2015b; see also Holmes et al., 2011) and are taken to evince that the absence of haptic feedback (i.e., BPH− trials) renders pantomime-grasps selectively mediated via relative visual information (see also Goodale et al., 1994; Westwood et al., 2000; Cavina-Pratesi et al., 2011; Fukui and Inui, 2013; Holmes et al., 2013). In turn, that haptic feedback provided immediately at the movement goal location (i.e., MG trials) or when experimentally induced (i.e., BPH+ trials) resulted in a violation of Weber’s law indicates an absolute visuo-haptic calibration. Moreover, in accounting for the integration hypothesis we emphasize that object size was randomly varied on a trial-by-trial basis. Thus, during BPH+ (and MG) trials it was not possible for participants to use haptic feedback from trial N-1 in order to support aperture scaling on a subsequent trial. Instead, we propose that an error signal related to the difference between a predicted and an actual haptic outcome activates a learning corrective process supporting the refinement and calibration of an internal forward model (see Flanagan et al., 2006). The internal model is proposed to mediate a visuo-haptic calibration serving the absolute specification of object size on future trial performances (see also Davarpanah Jazi and Heath, 2014; Whitwell et al., 2014).

### Blocked vs. Random Haptic Feedback Schedule: Preparing for the “Worst Case”

Recall that the objective of this study was to determine whether advanced information related to haptic feedback availability influences the nature of the information supporting PH− and PH+ trials. In addressing this objective, we note that previous work by Bingham et al. (2007) showed that PH− trials performed in a blocked feedback schedule exhibited smaller PGAs (and terminal grip apertures) than trials wherein the object was available to grasp at the movement goal location (i.e., H+ trials). In contrast, PH− trials in a random feedback schedule exhibited PGAs that were as large as blocked and random feedback schedule H+ trials. Accordingly, the authors proposed that intermittent—and unpredictable—terminal haptic feedback (i.e., random H+ trials) is sufficient to support absolute calibration in PH− trials. As outlined in the “Introduction” Section however, it could be argued that the larger PGAs associated with random feedback schedule PH− trials reflects a strategy designed to avoid the possibility of a hand/object collision. To avoid that potential confound, we contrasted PH− and PH+ trials in a random feedback schedule (i.e., RPH+ and RPH− trials) to preclude expectancy-based differences in grasping control. To that end, we found that the timing and magnitude of PGA, and the magnitude of TGA, for RPH− and RPH+ trials was equivalent to their blocked feedback schedule counterparts (i.e., BPH− and BPH+ trials). As such, the PGA findings demonstrate that the pantomime-grasping trial-types used here were associated with comparable control strategies, and our results provide no evidence that intermittent and unpredictable haptic feedback supports an absolute visuo-haptic calibration.

As indicated previously, JNDs (computed at PGA and TGA) for BPH− and BPH+ trials respectively adhered to and violated Weber’s law. In contrast, RPH− *and* RPH+ trials adhered to the law. Moreover, JND/object size slopes for RPH− and RPH+ trials did not reliably differ in magnitude from BPH− trials. That RPH− *and* RPH+ trials adhered to Weber’s law on par to BPH− trials provides law-based evidence that the inability to predict haptic feedback availability precluded an absolute calibration process and rendered aperture shaping via relative visual information. Thus, an important issue to address is why advance knowledge of haptic feedback is required to support an absolute visuo-haptic calibration. In addressing this question, we have drawn on work contrasting reaching/grasping movements performed with (i.e., closed-loop action) and without (i.e., open-loop) continuous limb and target vision across blocked and random feedback schedules. In particular, results have shown that closed-loop trials performed in a blocked feedback schedule are more accurate (Zelaznik et al., 1983; Elliott and Allard, 1985), exhibit more online trajectory amendments (Khan et al., 2002) and produce more effective PGAs (Jakobson and Goodale, 1991) than counterparts performed in a random feedback schedule. Accordingly, the inability to predict the availability of visual feedback has been interpreted to reflect the adoption of a “worst-case” control strategy wherein a response is specified largely in advance of movement execution via central planning mechanisms (Elliott et al., 2009). As well, work has shown that closed-loop reaching (Neely et al., 2008) and grasping (Heath et al., 2004) responses in a blocked feedback schedule are refractory to the context-dependent (i.e., relative) features of pictorial illusions, whereas random feedback schedule counterparts are “tricked” in a direction consistent with the illusion’s perceptual effects. As such, a “worst-case” control strategy has been tied to motor output subserved via relative visual information (for review see Heath et al., 2011). Indeed, it is entirely possible that in a “worst-case” control strategy the unpredictable nature of feedback diminishes participants’ ability to contrast an expected to an actual *visual* outcome and therefore limits the efficiency and effectiveness of an internal forward model supporting trial-by-trial performance improvements (Cheng and Sabes, 2007). In the context of the current investigation, an internal forward model would serve to trigger a learning corrective process when a mismatch is detected between a predicted and actual haptic outcome (Westling and Johansson, 1987). Thus, the predicted availability of haptic feedback (BPH+ trials) may represent the environment necessary for an optimal integration between visual and haptic systems (Ernst and Banks, 2002) and therefore supports the trial-by-trial learning corrective process required for an absolute visuo-haptic calibration. In contrast, we propose that completing a response in an environment wherein haptic feedback is unavailable (i.e., BPH− trials) or cannot be predicted (i.e., RPH− and RPH+ trials) limits—or precludes—an optimal integration process and results in motor output specified via the relative visual features of a target object.

## Conclusions

This work provides the first examination of whether pantomime-grasping performed with and without advance knowledge of haptic feedback adheres to or violates the psychophysical principles of Weber’s law. Results showed that grasping adhered to Weber’s law when haptic feedback was unavailable or could not be predicted—a finding we interpret to reflect the selective use of relative visual cues for aperture shaping. In contrast, responses violated Weber’s law when haptic feedback was predictably available. As such, we propose that trial-to-trial knowledge of haptic feedback serves as an optimal environment to support an absolute visuo-haptic calibration. Moreover, we again emphasize that our work identifies a critical limitation of the only other study to have examined the role of haptic feedback in a random feedback schedule (Bingham et al., 2007). Bingham et al.’s study is taken as explicit evidence for the sensory requirements associated with an absolute visuo-haptic calibration. Notably, and counter to Bingham et al. (2007), we show that advanced knowledge of haptic feedback is required to support an absolute haptic feedback calibration/integration. We therefore see our results as an important contribution to the grasping literature. Our future work in this area will examine the concurrent behavioral and electroencephalographic (i.e., event-related brain potentials: ERP; see Heath et al., 2015b) properties of pantomime-grasping responses performed with and without haptic feedback. In particular, the P300 ERP waveform is a component of interest because it reflects the updating of an internal mental model (Donchin and Coles, 1988). As such, modulation of the P300 amplitude in grasping paradigms similar to that used here would identify the neural mechanism associated with an absolute visuo-haptic calibration. Such a result would provide a more encompassing theoretical view of feedback in grasping, and may serve to emphasize its role and integration in future prosthetic and robotic interfaces.

## Author Contributions

SDJ was involved in designing and setting up the experiment as well as data collection. Further, she was involved in analyzing and explaining the findings of the study. MH supervised data collection and was involved in interpretation of findings. SDJ and MH contributed in writing the manuscript.

## Conflict of Interest Statement

The authors declare that the research was conducted in the absence of any commercial or financial relationships that could be construed as a potential conflict of interest.

## Footnotes

^1^The visual and tactile information mediating perceptions and actions differ with respect to their metrical properties. The term “relative” used for perceptions refers to target features (i.e., size, shape, and location) in relation to its surrounding environment and the manner in which it is encoded (e.g., the object is judged to be bigger or smaller than another object). Relative information is based on comparisons within the environment or memory-based experiences. In contrast, absolute information reflects the metrical (or Euclidean) properties of a target object. Notably, the use of “relative” and “absolute” information is reflected in the visual (Goodale, 2011) and somatosensory (i.e., tactile; see Lederman and Klatzky, 2009) literature.

^2^Although Schenk’s (2012) intermittent feedback schedule included randomly ordered H+ and PH− trials, participants were provided advanced knowledge of whether a trial would involve haptic feedback. Thus, and unlike the present study, Schenk’s work was not designed to evaluate whether the expectancy of haptic feedback influences an absolute visuo-haptic calibration.

^3^Although not provided in the main Results, JND/object size slopes computed at TGA match those computed at PGA. In particular, the slope for BPH− trials (0.09, SD = 0.06) was steeper than MG (0.00, SD = 0.05) and BPH+ (0.02, SD = 0.05) trials, (all *t*_(15)_ = 2.50 and 4.66, all *p* < 0.03). In turn, slopes for RPH− (0.07, SD = 0.07), RPH+ (0.07, SD = 0.06) and BPH− trials did not reliably differ from one another (all *t*_(15)_ = 1.22, 1.01 and −0.15, all *p* > 0.24, respectively for RPH− vs. BPH−, RPH+ vs. BPH−, and RPH− vs. RPH+).

^4^An issue raised in the review process was that the larger PGAs associated with MG trials in combination with the condition’s null scaling of JNDs relates to a mechanical constraint and/or the neuromotor noise accompanying a larger aperture opening. In addressing this issue, we first note that BPH+ trials elicited PGAs that matched the other pantomime-grasping conditions and were smaller than MG trials. In spite of this result, JNDs for BPH+ trials did not vary with object size. Moreover, all trial-types produced comparable TGA values. Thus, a mechanical-based explanation cannot account for the null JND/object size scaling observed at PGA and TGA for the MG and BPH+ trials. Second, a previous study by our group (Heath et al., 2012) was purpose-designed to examine if JND/object size scaling is related to the stochastic properties of motor output variability (for review see Schmidt et al., 1979). In that study participants completed grasps in movement time criterion of 400 ms and 800 ms. It was hypothesized that if impulse-variability impacts JND/object size scaling then grasping responses requiring higher velocities (i.e., 400 ms) would be associated with larger JND/object size slopes. Counter to that prediction, JND/object size slopes were refractory to the movement time criterion. Accordingly, our group proposed that the scaling of JNDs to object size is independent of neuromotor noise.

## References

[B1] Andersen R. A. Andersen K. N. Hwang E. J. Hauschild M. (2014). Optic ataxia: from Balint’s syndrome to the parietal reach region. Neuron 81, 967–983. 10.1016/j.neuron.2014.02.025 24607223 PMC4000741

[B2] Bingham G. Coats R. Mon-Williams M. (2007). Natural prehension in trials without haptic feedback but only when calibration is allowed. Neuropsychologia 45, 288–294. 10.1016/j.neuropsychologia.2006.07.011 17045314

[B3] Brainard D. H. (1997). The Psychophysics Toolbox. Spat. Vis. 10, 433–436. 10.1163/156856897x00357 9176952

[B4] Cavina-Pratesi C. Connolly J. D. Milner A. D. (2013). Optic ataxia as a model to investigate the role of the posterior parietal cortex in visually guided action: evidence from studies of patient M.H. Front. Hum. Neurosci. 7:336. 10.3389/fnhum.2013.00336 23882200 PMC3712225

[B5] Cavina-Pratesi C. Kuhn G. Ietswaart M. Milner A. D. (2011). The magic grasp: motor expertise in deception. PloS one 6:e16568. 10.1371/journal.pone.0016568 21347416 PMC3036651

[B6] Cheng S. Sabes P. N. (2007). Calibration of visually guided reaching is driven by error-corrective learning and internal dynamics. J. Neurophysiol. 97, 3057–3069. 10.1152/jn.00897.2006 17202230 PMC2536620

[B7] Cumming G. (2013). Understanding the New Statistics: Effect Sizes, Confidence Intervals and Meta-Analysis. New York: Routledge.

[B8] Davarpanah Jazi S. Heath M. (2014). Weber’s law in tactile grasping and manual estimation: feedback-dependent evidence for functionally distinct processing streams. Brain Cogn. 86, 32–41. 10.1016/j.bandc.2014.01.014 24556320

[B9] Davarpanah Jazi S. Hosang S. Heath M. (2015a). Memory delay and haptic feedback influence the dissociation of tactile cues for perception and action. Neuropsychologia 71, 91–100. 10.1016/j.neuropsychologia.2015.03.018 25796409

[B10] Davarpanah Jazi S. Yau M. Westwood D. A. Heath M. (2015b). Pantomime-grasping: the ’return’ of haptic feedback supports the absolute specification of object size. Exp. Brain Res. 233, 2029–2040. 10.1007/s00221-015-4274-0 25869741

[B11] Desmurget M. Epstein C. M. Turner R. S. Prablanc C. Alexander G. E. Grafton S. T. (1999). Role of the posterior parietal cortex in updating reaching movements to a visual target. Nat. Neurosci. 2, 563–567. 10448222 10.1038/9219

[B12] Donchin E. Coles M. G. H. (1988). Is the P300 component a manifestation of context updating? Behav. Brain Sci. 11, 357–427. 10.1017/s0140525x00058027

[B13] Elliott D. Allard F. (1985). The utilization of visual feedback information during rapid pointing movements. Q. J. Exp. Psychol. 37, 407–425. 10.1080/14640748508400942 4048546

[B14] Elliott D. Hansen S. Grierson L. E. (2009). Optimising speed and energy expenditure in accurate visually directed upper limb movements. Ergonomics 52, 438–447. 10.1080/00140130802707717 19401895

[B15] Ernst M. O. Banks M. S. (2002). Humans integrate visual and haptic information in a statistically optimal fashion. Nature 415, 429–433. 10.1038/415429a 11807554

[B16] Flanagan J. R. Bowman M. C. Johansson R. S. (2006). Control strategies in object manipulation tasks. Curr. Opin. Neurobiol. 16, 650–659. 10.1016/j.conb.2006.10.005 17084619

[B17] Fukui T. Inui T. (2013). How vision affects kinematic properties of pantomimed prehension movements. Front. Psychol. 4:44. 10.3389/fpsyg.2013.00044 23404470 PMC3566380

[B18] Ganel T. Chajut E. Algom D. (2008a). Visual coding for action violates fundamental psychophysical principles. Curr. Biol. 18, R599–R601. 10.1016/j.cub.2008.04.052 18644333

[B19] Ganel T. Chajut E. Tanzer M. Algom D. (2008b). Response: When does grasping escape Weber’s law? Curr. Biol. 18, R1090–R1091. 10.1016/j.cub.2008.10.007

[B20] Ganel T. Freud E. Chajut E. Algom D. (2012). Accurate visuomotor control below the perceptual threshold of size discrimination. PLoS One 7:e36253. 10.1371/journal.pone.0036253 22558407 PMC3338698

[B21] Geschwind N. Kaplan E. (1962). A human cerebral deconnection syndrome. A preliminary report. Neurology 12, 675–685. 10.1212/wnl.12.10.675 13898109

[B22] Goodale M. A. (2011). Transforming vision into action. Vision Res. 51, 1567–1587. 10.1016/j.visres.2010.07.027 20691202

[B23] Goodale M. A. Milner D. A. (2006). Sight Unseen: An Exploration of Conscious and Unconscious Vision. Oxford: Oxford University Press.

[B24] Goodale M. A. Jakobson L. S. Keillor J. M. (1994). Differences in the visual control of pantomimed and natural grasping movements. Neuropsychologia 32, 1159–1178. 10.1016/0028-3932(94)90100-7 7845558

[B25] Heath M. Davarpanah Jazi S. Holmes S. A. (2015a). An inverse grip starting posture gives rise to time-dependent adherence to Weber’s law: a reply to Ganel et al. (2014). J. Vis. 15:6. 10.1167/15.6.1 26024449

[B26] Heath M. Hassall C. D. MacLean S. Krigolson O. E. (2015b). Event-related brain potentials during the visuomotor mental rotation task: the contingent negative variation scales to angle of rotation. Neuroscience 311, 153–165. 10.1016/j.neuroscience.2015.10.018 26477986

[B27] Heath M. Holmes S. A. Mulla A. Binsted G. (2012). Grasping time does not influence the early adherence of aperture shaping to Weber’s law. Front. Hum. Neurosci. 6:332. 10.3389/fnhum.2012.00332 23267323 PMC3527824

[B28] Heath M. Mulla A. Holmes S. A. Smuskowitz L. R. (2011). The visual coding of grip aperture shows an early but not late adherence to Weber’s law. Neurosci. Lett. 490, 200–204. 10.1016/j.neulet.2010.12.051 21194553

[B29] Heath M. Neely K. A. Krigolson O. Binsted G. (2010). “Memory-guided reaching: what the visuomotor system knows and how long it knows it”, in Vision and Goal-directed Movement: Neurobehavioral Perspectives, eds Elliott D. Khan M. (Champaign, IL: Human Kinetics), 79–96.

[B30] Heath M. Rival C. Binsted G. (2004). Can the motor system resolve a premovement bias in grip aperture? Online analysis of grasping the Müller-Lyer illusion. Exp. Brain Res. 158, 378–384. 10.1007/s00221-004-1988-9 15278330

[B31] Holmes S. A. Heath M. (2013). Goal-directed grasping: the dimensional properties of an object influence the nature of the visual information mediating aperture shaping. Brain Cogn. 82, 18–24. 10.1016/j.bandc.2013.02.005 23501700

[B32] Holmes S. A. Lohmus J. McKinnon S. Mulla A. Heath M. (2013). Distinct visual cues mediate aperture shaping for grasping and pantomime-grasping tasks. J. Motor. Behav. 45, 431–439. 10.1080/00222895.2013.818930 23971991

[B33] Holmes S. A. Mulla A. Gordon B. Heath M. (2011). Visually and memory-guided grasping: aperture shaping exhibits a time-dependent scaling to Weber’s law. Vision Res. 51, 1941–1948. 10.1016/j.visres.2011.07.005 21777599

[B34] Hosang S. Chan J. Davarpanah Jazi S. Heath M. (2016). Grasping a 2D object: terminal haptic feedback supports an absolute visuo-haptic calibration. Exp. Brain Res. 234, 945–954. 10.1007/s00221-015-4521-4 26680769

[B35] Jakobson L. S. Goodale M. A. (1991). Factors affecting higher-order movement planning: a kinematic analysis of human prehension. Exp. Brain Res. 86, 199–208. 10.1007/bf00231054 1756790

[B36] James T. W. Culham J. C. Humphrey G. K. Milner A. D. Goodale M. A. (2003). Ventral occipital lesions impair object recognition but not object-directed grasping: an fMRI study. Brain 126, 2463–2475. 10.1093/brain/awg248 14506065

[B37] Jeannerod M. (1986). Mechanisms of visuomotor coordination: a study in normal and brain-damaged subjects. Neuropsychologia 24, 41–78. 10.1016/0028-3932(86)90042-4 3517680

[B38] Khan M. A. Elliot D. Coull J. Chua R. Lyons J. (2002). Optimal control strategies under different feedback schedules: kinematic evidence. J. Mot. Behav. 34, 45–57. 10.1080/00222890209601930 11880249

[B39] Lederman S. J. Klatzky R. L. (2009). Haptic perception: a tutorial. Atten. Percept. Psychophys. 71, 1439–1459. 10.3758/app.71.7.1439 19801605

[B40] Lemay M. Proteau L. (2001). A distance effect in a manual aiming task to remembered targets: a test of three hypotheses. Exp. Brain Res. 140, 357–368. 10.1007/s002210100834 11681311

[B41] Liepmann H. (1905/1980). The left hemisphere and action (Translation from Münchener Medizinische Wochenschriff 1905, 48–49) Translations from Liepmann’s essays on apraxia. Research Bulletin 506, Department of Psychology, University of Western Ontario, London

[B42] Loftus G. R. Masson M. E. J. (1994). Using confidence intervals in within-subject designs. Psychon. Bull. Rev. 1, 476–490. 10.3758/bf03210951 24203555

[B43] Marks L. E. Algom D. (1998). “Psychophysical scaling”, in Measurement, Judgement and Decision Making, ed. M. H. Birnbaum (San Diego: Academic Press), 81–178.

[B44] Milgram P. (1987). A spectacle-mounted liquid-crystal tachistoscope. Behav. Res. Methods Instrum. Comput. 19, 449–456. 10.3758/bf03205613

[B45] Milner A. D. Ganel T. Goodale M. A. (2012). Does grasping in patient D.F. depend on vision? Trends Cogn. Sci. 16, 256–257; discussion 258–259. 10.1016/j.tics.2012.03.004 22425666

[B46] Neely K. A. Tessmer A. Binsted G. Heath M. (2008). Goal-directed reaching: movement strategies influence the weighting of allocentric and egocentric visual cues. Exp. Brain Res. 186, 375–384. 10.1007/s00221-007-1238-z 18087697

[B47] Pedhazur E. J. (1997). Multiple Regression in Behavioral Research: Explanation and Prediction Orlando: Harcourt Brace College Publisher.

[B48] Pettypiece C. E. Culham J. C. Goodale M. A. (2009). Differential effects of delay upon visually and haptically guided grasping and perceptual judgments. Exp. Brain Res. 195, 473–479. 10.1007/s00221-009-1807-4 19404627

[B49] Pisella L. Grea H. Tilikete C. Vighetto A. Desmurget M. Rode G.. (2000). An ‘automatic pilot’ for the hand in human posterior parietal cortex: toward reinterpreting optic ataxia. Nat. Neurosci. 3, 729–736. 10.1038/76694 10862707

[B50] Roy E. A. Heath M. Westwood D. Schweizer T. A. Dixon M. J. Black S. E.. (2000). Task demands and limb apraxia in stroke. Brain Cogn. 44, 253–279. 10.1006/brcg.2000.1230 11041991

[B51] Schenk T. (2012). No dissociation between perception and action in patient DF when haptic feedback is withdrawn. J. Neurosci. 32, 2013–2017. 10.1523/jneurosci.3413-11.2012 22323715 PMC6621711

[B52] Schmidt R. A. Zelaznik H. Hawkins B. Frank J. S. Quinn J. T. (1979). Motor-output variability: a theory for the accuracy of rapid motor acts. Psychol. Rev. 47, 415–451. 10.1037/0033-295x.86.5.415 504536

[B53] Smeets J. B. J. Brenner E. (1999). A new view on grasping. Motor Control 3, 237–271. 10409797 10.1123/mcj.3.3.237

[B54] Westling G. Johansson R. S. (1987). Responses in glabrous skin mechanoreceptors during precision grip in humans. Exp. Brain Res. 66, 128–140. 10.1007/bf00236209 3582527

[B55] Westwood D. A. Chapman C. D. Roy E. A. (2000). Pantomimed actions may be controlled by the ventral visual stream. Exp. Brain Res. 130, 545–548. 10.1007/s002219900287 10717797

[B56] Whitwell R. L. Buckingham G. (2013). Reframing the action and perception dissociation in DF: haptics matters, but how? J. Neurophysiol. 109, 621–624. 10.1152/jn.00396.2012 22855778

[B57] Whitwell R. L. Milner A. D. Cavina-Pratesi C. Byrne C. M. Goodale M. A. (2014). DF’s visual brain in action: the role of tactile cues. Neuropsychologia 55, 41–50. 10.1016/j.neuropsychologia.2013.11.019 24300664

[B58] Winter D. A. Patla A. E. (1997). Signal Processing and Linear Systems for the Movement Sciences. Waterloo: St Waterloo Biomechanics

[B59] Wolpert D. M. Ghahramani Z. Jordan M. I. (1995). An internal model for sensorimotor integration. Science 269, 1880–1882. 10.1126/science.7569931 7569931

[B60] Zelaznik H. Z. Hawkins B. Kisselburgh L. (1983). Rapid visual feedback processing in single-aiming movements. J. Mot. Behav. 15, 217–236. 10.1080/00222895.1983.10735298 15151871

